# Prevalence of feeding difficulties in children aged six months to six years who were born prematurely

**DOI:** 10.1590/2317-1782/e20240194en

**Published:** 2025-03-31

**Authors:** Cícero Alaor Kluppel, Amanda Bencke Teixeira da Silva, Daniel Boquai Camargo, Adriane Celli, Ana Lúcia Figueiredo Sarquis

**Affiliations:** 1 Departamento de Pediatria, Complexo Hospital de Clínicas, Universidade Federal do Paraná – UFPR - Curitiba (PR), Brasil.; 2 Programa de Pós-graduação em Saude da Criança e Adolescência, Departamento de Pediatria do Complexo, Hospital de Clínicas – CHC, Universidade Federal do Paraná – UFPR - Curitiba (PR), Brasil.

**Keywords:** Avoidant Restrictive Food Intake Disorder, Food Fussiness, Infant, Premature, Child Nutrition

## Abstract

**Purpose:**

To describe the prevalence of feeding difficulties in preterm children aged six months to six years and eleven months, and to analyze the relationships with perinatal and neonatal conditions.

**Methods:**

This cross-sectional ambispective study applied the Brazilian Infant Feeding Scale to the parents of 129 children followed in preterm outpatient clinics to assess the prevalence of feeding difficulties. Additional variables were collected retrospectively from medical records.

**Results:**

Fifteen children (11.62%) out of 129 exhibited feeding difficulties. Significant influencing variables were being born small for gestational age, having a mother with gestational diabetes mellitus, and undergoing phototherapy. Ventilatory support duration correlated with the Motor-Oral domain, and phototherapy duration correlated with the Sensory-Oral domain of the Brazilian Infant Feeding Scale.

**Conclusion:**

The Brazilian Infant Feeding Scale showed that the prevalence of long-term Feeding Difficulty in preterm infants was 11.62%. Small for Gestational Age newborns showed a higher prevalence. Children undergoing phototherapy and offspring of mothers with gestational diabetes showed a lower prevalence. The other variables studied did not significantly affect the prevalence of Feeding Difficulties, but the duration of ventilatory support affected the Oral-motor domain, and the duration of phototherapy also affected the Oral-Motor. This study marks the first application of the Brazilian Infant Feeding Scale in Brazilian preterm infants.

## INTRODUCTION

Feeding Difficulties (FD) are common complaints among parents, affecting 19% to 50% of children^(1)^. In 2015, Kerzner et al.^(2)^ proposed the term Feeding Difficulty as a standard for childhood feeding problems. This study suggests that whenever a mother says, " if the mother says there’s a problem, there is a problem," the attending professional should look for biopsychosocial signs of severity and assess the need for treatment.

In addition to issues such as low weight, delays in the development of orofacial motor skills, and other nutritional deficiencies, FD can lead to long-term cognitive and behavioral problems, such as neurodevelopmental deficits, eating disorders, fear of eating with others, and obsessive-compulsive symptoms^(3)^.

The population of preterm infants is more susceptible to FD due to perinatal and neonatal factors such as immaturity, very low weight, neurological complications, prolonged nasogastric tube feeding, parenteral nutrition, extended ventilatory support, among others, in addition to the higher frequency of gastroesophageal reflux and other associated medical conditions^(4,5)^. These factors contribute to these children presenting disorganized or dysfunctional feeding patterns, such as oral motor dysfunction, and, in the long term, developing persistent feeding problems with nutritional impact and practical challenges in daily routines, as well as emotional burdens for their families^(4,6,7)^.

Premature infants are also more prone to sensory processing problems^(8)^, characterized by deficits in perceiving, interpreting, or modulating sensory stimuli of visual, tactile, auditory, vestibular, proprioceptive, gustatory, and/or olfactory nature. These sensory deficits can influence how the child perceives and reacts to stimuli related to feeding^(9)^.

Given the potential short- and long-term nutritional and psychosocial problems, the diagnosis of FD is essential to identify the specific needs of each child in the medical, nutritional, psychosocial, and developmental domains, enabling timely and appropriate interventions to prevent health complications and improve the quality of life for the child and their family^(10)^.

To diagnose FD, several instruments are available with a wide range of heterogeneity: some based on questionnaires, others on direct swallowing observation, evaluating aspects ranging from oral sensitivity to the child's oral motor skills^(11)^. The *Escala Brasileira de Alimentação Infantil – (EBAI)* (Brazilian Infant Feeding Scale), a screening instrument that allows comprehensive evaluation of the biopsychosocial dimensions of FD, is a validated cross-cultural tool comprising 14 self-administered questions. It evaluates FD severity and seven domains, namely: oral motor, sensory oral, appetite, and four others related to psychosocial conditions (maternal concern about feeding, child behavior during meals, maternal strategies during meals, and family reactions to the child's feeding). Due to these characteristics, *EBAI* is a quick and useful tool for professionals wishing to screen newborns for FD^(12)^.

This study aims to describe the prevalence of feeding difficulties in children aged six months to six years and eleven months, born preterm, and analyze their relationship with the investigated perinatal and neonatal conditions.

## METHODS

This is a cross-sectional ambispective study, approved by the Research Ethics Committee of the institution under *CAAE* number: 65887622.1.0000.0096.

**Figure 1:**
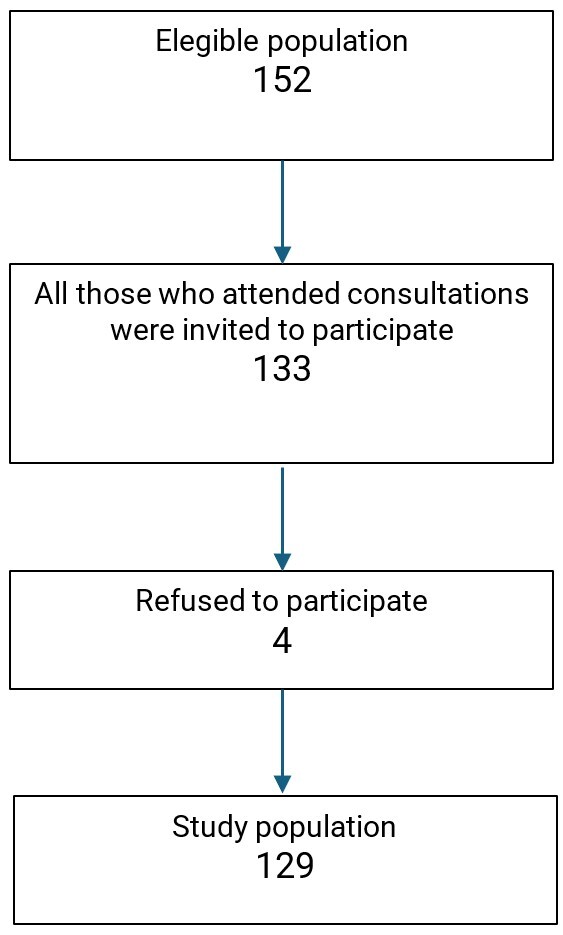
Study Population

The research involved 129 parents/caregivers of preterm children, fed with solid or semi-solid diets, who signed the Informed Consent Form during consultations at the preterm outpatient clinics of a tertiary hospital, from February 2023 to January 2024. In the clinic, 152 eligible children were followed, and the sample calculation indicated that, with a 95% confidence level and a 5% margin of error, the minimum sample size would be 110 individuals. A total of 129 children were conveniently selected (Figure 1). None of the participating children had received speech therapy treatment.

Preterm infants were defined as children born before 37 weeks of gestational age, determined by early ultrasound^(13)^ or the New Ballard method^(14)^, and classified into four groups: late preterm (34 weeks to 36 weeks and 6 days), moderate preterm (32 weeks to 33 weeks and 6 days), very preterm (28 weeks to 31 weeks and 6 days), and extremely preterm (< 28 weeks)^(15,16)^. The study population consisted of children aged six months corrected age to five years and seven months.

After agreement, caregivers were invited to self-complete the *EBAI* ^(12)^. Perinatal and neonatal data were collected from medical records. The variables obtained included: child’s sex, gestational age, age at the time of application, birth weight, chronological age at discharge, duration of orotracheal intubation (OTI), days of ventilatory support, duration of phototherapy, degree of prematurity, gestational diabetes (GDM), Hypertensive Disorder of Pregnancy (HDP), type of delivery, APGAR scores at the first and fifth minutes, presence of jaundice, and birth weight for gestational age according to the Fenton growth chart^(17)^ – Small for Gestational Age (SGA), Appropriate for Gestational Age (AGA), and Large for Gestational Age (LGA)^(18)^.

Inclusion criteria considered families with children aged six months to six years and eleven months corrected age, born preterm, evaluated by early ultrasound (before 13 weeks and six days of gestation or, in its absence, by the New Ballard method) and caregiver’s agreement to participate in the study.

Exclusion criteria included complex congenital malformations, use of alternative feeding routes, absence of dietary introduction, non-neurotypical children, and missing critical data in medical records (such as birth weight and gestational age).

### *Escala Brasileira de Alimentação Infantil (EBAI)* (Brazilian Infant Feeding Scale)

*EBAI* is a screening tool comprising 14 questions, easily and quickly completed by parents (self-administered). It was cross-culturally validated for Brazil in 2021^(12)^ from the Montreal Children’s Hospital Feeding Scale (MCH-FS) ^(19)^. *EBAI* completion results are classified into four categories: no FD, mild FD, moderate FD, and severe FD. The 14 items cover seven overlapping domains: oral motor (items 8 and 11), sensory oral (items 7 and 8), appetite (items 3 and 4), maternal concern about feeding (items 1, 2, and 12), behavior during meals (items 6 and 8), maternal strategies (items 5, 9, and 10), and family reactions to feeding (items 13 and 14), the latter four domains comprising the psychosocial dimension. Each item is scored on a seven-point Likert scale. Seven items range from negative to positive (with 1 being more severe and 7 being problem-free), and seven range from positive to negative. The mother or caregiver marks each item according to the frequency of occurrence, perceived difficulty of a behavior, or the level of concern about the question. After summing the scores, a Raw Score is obtained, converted into a T-score table, with scores equal to or greater than 61 indicating a positive FD screening. The questionnaire can be completed in approximately five minutes. For scoring, items ranging from negative to positive are inverted by the researcher, who calculates the raw score and converts it into a T-score table, classifying FD into four categories^(12)^.

### Statistical Analysis

Data were organized in an Excel® spreadsheet and analyzed using IBM SPSS Statistics v.29.0. Results of quantitative variables were described by mean, standard deviation, median, minimum, and maximum. Categorical variables (sex, degree of prematurity, GDM, SHDP, type of delivery, categorized APGAR score, jaundice, and weight for gestational age) were described by absolute and percentage frequency. Comparisons between two groups defined by FD-related variables (no or yes) and by type of delivery (vaginal or cesarean), regarding quantitative variables (gestational age, age at questionnaire application, birth weight, age at discharge, duration of OTI, days of ventilatory support, and phototherapy duration), were performed using the Student’s t-test for independent samples or the Mann-Whitney non-parametric test. Groups defined by prematurity (late, moderate, very preterm, or extreme) and by AGA/SGA/LGA were compared regarding quantitative variables using the Kruskal-Wallis non-parametric test and Dunn’s post-hoc test. Categorical variables were analyzed using Fisher’s exact test or Chi-square test. To evaluate the correlation between two quantitative variables, Spearman correlation coefficients were estimated. The normality condition of quantitative variables was assessed by the Kolmogorov-Smirnov test. P-values < 0.05 indicated statistical significance. For multiple group comparisons, p-values were Bonferroni corrected.

## RESULTS

In the population of 129 children, 52.7% were female and 47.3% male. Birth weights ranged from 540g to 3585g. Most individuals were classified as Late Preterm, followed in number by Very Preterm, Moderate Preterm, and, in smaller numbers, Extremely Preterm (Table 1). At the time of application, the average age of children was 1.8 years, with a median of 1.7 years (minimum of 0.6 years and maximum of 5.6 years).

**Table 1:** Relationship between perinatal and neonatal variables and feeding difficulties

**Variable**	**Classification**	**n**	**Group**		**p** ^*^	**OR (CI95%)**
			**Normal**	**Feeding Difficulty**		
BIRTH WEIGHT FOR GESTATIONAL AGE	LGA (ref)	7	7 (100%)	0 (0%)		
	AGA	95	87 (91.6%)	8 (8.4%)	00.300	-
	SGA	27	20 (74.1%)	7 (25.9%)	00.022	-
GDM	No	102	87 (83%)	15 (14.7%)		
	Yes	27	27 (100%)	0 (0%)	00.040	-
Phototherapy	No	40	30 (75%)	10 (25%)		
	Yes	88	83 (94.3%)	5 (5.7%)	00.004	0,18 (0.06 – 0.57)
Jaundice	No	29	23 (79.3%)	6 (20.7%)		
	Yes	99	90 (90.9%)	9 (9.1%)	00.096	0.38 (0.12 – 1.19)
Age at *EBAI* application (years)	[mean ± SD (min-max)]	129	1.8 ± 0,9	1.9 ± 0.8	00,587	1,16 (0.68 – 2.00)
			(0.6 – 5.6)	(0.7 – 4.3)		
Days hospitalized	[mean ± SD (min-max)]	129	30.5	23	00.598	1.00 (0.98 – 1.01)
			(3 - 173)	(3 - 113)		
GA (weeks)	[mean ± SD (min-max)]	129	32 ± 3.2	33 ± 2.7	00.282	1.11 (0.92 – 1.34)
			(24 – 36.9)	(25.6 – 36.7)		
Birth weight (g)	[mean ± SD (min-max)]	129	1705 ± 667	1599 ± 562	00.558	1 (0.999 – 1.001)
			(540 - 3585)	(859 - 2830)		
Duration of intubation (OI) (days)	[mean ± SD (min-max)]	129	0 (0 - 48)	0 (0 - 17)	00.391	0,95 (0.85 – 1.06)
Total days with respiratory support	[mean ± SD (min-max)]	129	5 (0 - 107)	3 (0 - 85)	00.810	1 (0.97 – 1.02)
Duration of phototherapy (days)	[mean ± SD (min-max)]	128	2 (0 - 10)	0 (0 - 5)	00.040	0.63 (0.40 – 0.98)
Prematurity	Late Preterm (ref)	44	39 (88.6%)	5 (11.4%)		
	Moderate Preterm	32	25 (78.1%)	7 (21.9%)	00.222	2.18 (0.62 – 7.64)
	Very Preterm	36	34 (94.4%)	2 (5.6%)	00.370	0.46 (0.08 – 2.52)
	Extreme Preterm	17	16 (94.1%)	1 (5.9%)	00.527	0.49 (0.05 – 4.51)
HDP	No	94	86 (91.5%)	8 (8.5%)		
	Yes	34	27 (79.4%)	7 (20.6%)	00.068	2.79 (0.93 – 8.40)
MODE OF DELIVERY	Vaginal	44	42 (95.5%)	2 (4.5%)		
	Cesarean	83	70 (84.3%)	13 (15.7%)	00.083	3.90 (0.84 – 18.1)
Apgar score at 1 min	(7 –10) (ref)	80	68 (85.0%)	12 (15.0%)		
	(4 – 6)	37	35 (94.6%)	2 (5.4%)	00.154	0.32 (0.07 – 1.53)
	(0 – 3)	12	11 (91.7%)	1 (8.3%)	00.543	0.52 (0.06 – 4.37)
Apgar score at 5 min	(7 – 10)	117	103 (88.0%)	14 (12.0%)		
	(4 – 6)	11	10 (90.9%)	1 (9.1%)		
	(0 – 3)	1	1	0	-	-
Sex	Female	68	62 (91.2%)	6 (8.8%)		
	Male	61	52 (85.2%)	9 (14.8%)	00.283	1.80 (0.61 – 5.46)

p < 0.05

* Logistic regression model and Wald test or Fisher’s exact test

**Caption:** GDM = gestational diabetes mellitus; HDP = Hypertensive Disorders of Pregnancy; SGA = small for gestational age; AGA = appropriate for gestational age; LGA = large for gestational age; OI = orotracheal intubation; ref = reference variable; min = minute; g = gram; OR: odds ratio; 95% CI: 95% confidence interval; min = minimum value; max = maximum value; SD = Standard Deviation; GA = Gestational Age

### Prevalence of Feeding Difficulties in Preterm

Among the 129 participating children, 15 presented FD, indicating a prevalence of 11.62%. Of these, six (40%) had mild FD, seven (47.7%) moderate FD, and two (13.3%) severe FD.

### Relationship between perinatal and neonatal variables and FD

Only three independent variables studied showed a significant relationship with FD (p < 0.05): birth weight classification for gestational age, gestational diabetes mellitus (GDM), and phototherapy use. The remaining variables showed no statistical significance.

#### Birth weight classification for gestational age

In the studied population, 73.64% of children were AGA, 20.93% were SGA, and 5.42% were LGA. Among LGA, no child presented FD, while the highest incidence was observed in SGA.

The prevalence of FD in SGA is significantly higher than in other birth weight categories (Table 1).

Although the SGA population was smaller than the AGA population, nearly half of the children with FD were SGA. This proportion was statistically significant (Table 2).

**Table 2:** Proportion of individuals with and without GDM and Weight for Gestational Age

	n	SGA	AGA	LGA
No FD	114	20(17.5%)	87(76.4%)	7(6.1%)
FD	15	7 (46.7%)	8(53.3%)	0(0.0%)
TOTAL	129	27	95	7
		p=0.023	p=0.112	p=0.703

**Caption:** n=number of subjects; SGA=small for gestational age; AGA=appropriate for gestational age; LGA=large for gestational age; FD =Feeding Difficulty; .

#### Gestational Diabetes Mellitus (GDM) and phototherapy

Among children born to mothers with GDM, none presented FD. All children with FD were born to mothers without GDM, showing statistical significance.

FD prevalence was lower in those who underwent phototherapy, especially with longer phototherapy durations. This difference was statistically significant.

The duration of phototherapy was analyzed concerning the AGA, SGA, and LGA groups, revealing a significantly shorter duration in the SGA population compared to the other groups (Table 3).

**Table 3:** Phototherapy duration and birth weight for gestational age

**VARIABLE**	**AGA/SGA/LGA**	**n**	**MEAN**	**Standard Deviation**	**Median**	**Minimum**	**Maximum**	**p** ^*^
Phototherapy time(days)	AGA	94	2.1	2.1	2	0	10	
	SGA	27	1.0	1.4	0	0	5	
	LGA	7	2.9	2.8	2	0	8	0.014

p < 0.05

* Kruskal-Wallis non-parametric test

Spearman's correlation coefficient analysis showed a weak inverse correlation between phototherapy duration and all *EBAI* domains, with a significant inverse correlation in the sensory-oral domain. Conversely, the duration of ventilatory support showed a weak but significant direct correlation with the oral motor domain (Table 4). Other studied variables did not show statistical significance with any *EBAI* domains.

**Table 4:** Correlation between phototherapy duration and ventilatory support with the domains assessed in the *EBAI*

	n	r	p
Phototherapy time(days) *vs* T-score	128	-0.10	0.240
Phototherapy time (days) *vs* Oral motor	128	-0.05	0.573
Phototherapy time (days) *vs* Sensory oral	128	-0.25	0.005
Phototherapy time (days) *vs* Appetite	128	-0.12	0.172
Phototherapy time (days) *vs* Maternal concern about feeding	128	-0.17	0.053
Phototherapy time (days) *vs* Behavior during meals	128	-0.15	0.093
Phototherapy time (days) *vs* Maternal strategies	128	-0.05	0.543
Phototherapy time (days) *vs* Family reactions to feeding	128	-0.10	0.240
Days of ventilatory support *vs* T-score	129	0.14	0.125
Days of ventilatory support *vs* Oral motor	129	0.18	0.045
Days of ventilatory support *vs* Sensory oral	129	0.03	0.698
Days of ventilatory support *vs* Appetite	129	-0.01	0.908
Days of ventilatory support *vs* Maternal concern about feeding	129	0.05	0.585
Days of ventilatory support *vs* Behavior during meals	129	0.08	0.347
Days of ventilatory support *vs* Maternal strategies	129	-0.02	0.817
Days of ventilatory support *vs* Family reactions to feeding	129	0.14	0.125

**Caption:** n=number of individuals; r=Spearman's coefficient; n=number of individuals; T-score= result of the Brazilian Infant Feeding Scale score (*Escala Brasileira de alimentção Infantil-EBAI).*

No significant associations were found between other studied variables and FD.

## DISCUSSION

This study revealed that FD, assessed using the *EBAI*, is relatively low compared to most previous studies. A meta-analysis evaluating 22 studies with 4,381 preterm children across all gestational ages, utilizing various assessment methods such as formal, informal, or clinical scales up to 48 months of age, estimated that 42% of preterm children might present some type of FD, with no significant prevalence across gestational age categories or assessment age ^(5)^. A critical review of 22 studies involving 3,149 children, using structured questionnaires or direct observation, estimated that between 25% and 80% of preterm children might experience FD^(6)^.

In this study, the prevalence of FD was 11.62% (n=15), closely matching the 11% reported by Nieuwenhuis et al. using the Screeningslijst Eetgedrag Peuters (SEP) questionnaire, a Dutch-validated screening tool derived from the MCH-FS. In the same study, this author compared 30 preterm children with 248 non-preterm children at three years of age and found no significant differences between SGA preterm and non-preterm children^(20)^.

In this study, being born SGA was statistically significant in determining FD, with 25.92% of SGA individuals presenting FD compared to only 8.42% of AGA and none of the LGA.

SGA births, particularly when preterm, predispose children to neurological developmental issues, potentially increasing the risk of non-severe neurological dysfunction, cognitive and attention problems, and low social skills^(21)^. Mealtime interactions demand motor, sensory, and cognitive skills from the child while fostering interaction with caregivers^(10)^. Subtle neurodevelopmental consequences may favor the emergence of FD.

Children born to mothers with GDM showed no FD cases. Despite GDM being a predisposing factor for LGA births^(22)^, with rates ranging from 20% to 30%^(23)^, only two (7.40%) offspring of mothers with GDM were LGA. Most (74%) were AGA, possibly reflecting adequate prenatal management in a tertiary university hospital setting. Literature references on the negative relationship between being born preterm to a GDM mother and FD are scarce. Contrarily, studies highlight negative effects of maternal GDM on the offspring's physical health, neurodevelopment, and cognition^(24,25)^. The low prevalence of SGA in this group (18.6%), which showed the highest FD prevalence, may explain this finding.

Children subjected to phototherapy also exhibited fewer FD cases. The explanation for this finding remains unclear, and no literature supports a negative relationship between phototherapy and FD. However, extremely preterm infants exposed to phototherapy display less neurological impairment compared to their non-exposed counterparts^(26)^. Another plausible explanation is that FD prevalence was higher in SGA, who had significantly shorter phototherapy durations than AGA and LGA. Therefore, correlations might be confounded, as AGA and LGA reduce FD risk, and these groups underwent more phototherapy, creating an apparent inverse relationship between phototherapy duration and FD.

Given the lack of literature support, the negative relationship between GDM and phototherapy with FD requires further study, as it could be incidental.

Spearman's correlation coefficients revealed weak relationships among most variables and *EBAI* domains. Notably, phototherapy duration inversely but weakly correlated with various domains, significantly in the sensory-motor domain. Ventilatory support duration correlated weakly but significantly with worsening of the oral motor domain, supported by literature, though the finding lacks detailed explanation^(7,27)^.

Despite prematurity being cited as a cause of FD^(7)^, this study found no significant differences among prematurity grades. FD results from multiple factors, not prematurity alone^(28)^. Additionally, oral motor dysfunction frequency might decrease over time due to growth and psychomotor development in preterm infants^(27)^.

### Limitations

Most children in this study were at-risk preterm infants requiring intensive care and tertiary outpatient follow-up. Comparative studies with less severe preterm and non-preterm infants might yield different prevalence and risk/protection factor results.

Differences with existing literature may stem from methodological variations: direct feeding observation^(29)^, semi-structured parental interviews^(30)^, or using a different questionnaire than *EBAI* ^(31)^. Instruments often assess specific domains, whereas *EBAI*—a comprehensive screening tool—includes sensory, motor, appetite, and psychosocial aspects^(12)^.

Retrospective data retrieval from medical records sometimes faces standardization issues or missing information. A long-term prospective study with larger patient numbers could provide a better understanding of FD in preterm infants.

## CONCLUSION

Using a validated Brazilian instrument (*EBAI*), this study determined that the prevalence of FD in the examined preterm population is 11.62%. SGA preterm infants exhibited higher FD prevalence than AGA and LGA. Preterm infants subjected to phototherapy, those with longer phototherapy durations, and those born to GDM mothers exhibited fewer FD cases. Other perinatal and neonatal variables lacked statistical association with FD.
