# Supplementary material for: Prevalence of feeding difficulties in children aged six months to six years who were born prematurely
Source: Codas. 2025 Mar 31;37(3):e20240194. doi: 10.1590/2317-1782/e20240194en (PMC11963878; doi:10.1590/2317-1782/e20240194en)
Supplement: Translation

# Translation: Prevalência de dificuldades alimentares em crianças entre seis meses e seis anos que nasceram prematuras

**DOI:** 10.1590/2317-1782/e20240194pt

**Authors:** Cícero Alaor Kluppel, Amanda Bencke Teixeira da Silva, Daniel Boquai Camargo, Adriane Celli, Ana Lúcia Figueiredo Sarquis

**Affiliations:** 1 Departamento de Pediatria, Complexo Hospital de Clínicas, Universidade Federal do Paraná – UFPR - Curitiba (PR), Brasil.; 2 Programa de Pós-graduação em Saude da Criança e Adolescência, Departamento de Pediatria do Complexo, Hospital de Clínicas – CHC, Universidade Federal do Paraná – UFPR - Curitiba (PR), Brasil.

**Keywords:** Transtorno Alimentar Restritivo Evitativo, Seletividade Alimentar, Recém-nascido Prematuro, Nutrição da Criança

## Abstract

**Objetivo:**

Descrever a prevalência de dificuldades alimentares em crianças com idade entre seis meses e seis anos e onze meses, nascidas prematuras, e analisar as relações com as condições perinatais e neonatais.

**Método:**

Estudo ambispectivo transversal, no qual a Escala Brasileira de Alimentação Infantil foi aplicada aos pais de 129 crianças acompanhadas em ambulatórios de prematuros, para avaliar a prevalência de Dificuldade Alimentar. As demais variáveis foram coletadas retrospectivamente nos prontuários.

**Resultados:**

Quinze crianças (11,62%), das 129 que participaram, apresentaram Dificuldade Alimentar. As variáveis que influenciaram significativamente o resultado foram: nascer pequeno para a idade gestacional, ser filho de mãe com Diabetes Mellitus Gestacional e ser submetido à fototerapia. Ao observar os domínios avaliados pela Escala Brasileira de Alimentação infantil, foi possível observar que o tempo de suporte ventilatório teve correlação com o domínio Motor-Oral e o tempo de fototerapia com o domínio Sensório-Oral.

**Conclusão:**

A Escala Brasileira de Alimentação Infantil mostrou que a prevalência de Dificuldade Alimentar a longo prazo em nascidos prematuros foi de 11,62%. Nascidos pequenos para a idade gestacional apresentaram maior prevalência. Crianças submetidas à fototerapia e filhos de mães com diabetes gestacional apresentaram menor prevalência. As outras variáveis estudadas não afetaram significativamente a prevalência de Dificuldades Alimentares, mas o tempo de suporte ventilatório afetou o domínio Motor-Oral e o tempo de fototerapia o Motor-Oral. Este estudo pioneiro marca a primeira aplicação da Escala Brasileira de Alimentação Infantil em crianças brasileiras nascidas prematuras.

## INTRODUÇÃO

Dificuldades Alimentares (DA) são queixas frequentes dos pais, ocorrendo em 19% a 50% das crianças^(1)^. Kerzner et al.^(2)^ propuseram, em 2015, o termo Dificuldade Alimentar como padrão para os problemas alimentares da criança. Esse trabalho sugere que, sempre que uma mãe diz “meu filho não come”, o profissional assistente deverá procurar sinais biopsicossociais de gravidade e avaliar a necessidade de tratamento.

Além de problemas como baixo peso, atrasos no desenvolvimento da motricidade orofacial e outras deficiências nutricionais, as DA podem, a longo prazo, causar problemas cognitivos e comportamentais, como déficit do neurodesenvolvimento, transtornos alimentares, medo de comer com outras pessoas e sintomas obsessivos-compulsivos^(3)^.

A população de nascidos prematuros é mais suscetível à DA, em decorrência de fatores perinatais e neonatais como imaturidade, muito baixo peso, complicações neurológicas, tempo prolongado de sonda nasogástrica, nutrição parenteral, tempo de suporte ventilatório, entre outros fatores, além da maior frequência de refluxo gastroesofágico e outras condições médicas associadas^(4,5)^. Esses fatores fazem com que essas crianças apresentem padrões alimentares desorganizados ou disfuncionais, como disfunção da motricidade oral e, a longo prazo, desenvolvam problemas alimentares persistentes, com impacto nutricional e desafios práticos na rotina diária e emocionais para suas famílias^(4,6,7)^ .

Prematuros ainda estão mais sujeitos a problemas de processamento sensorial^(8)^, caracterizados por déficits em perceber, interpretar ou modular estímulos sensoriais de natureza visual, tátil, auditiva, vestibular, proprioceptiva, gustativa e/ou olfativa. Esses déficits sensoriais podem influenciar a forma como a criança percebe e reage aos estímulos relacionados à alimentação^(9)^.

Em virtude dos possíveis problemas nutricionais e psicossociais a curto e longo prazo, o diagnóstico das DA é importante para identificar necessidades específicas de cada criança nos domínios médico, nutricional, psicossocial e do desenvolvimento, para que intervenções adequadas e em tempo oportuno sejam implementadas a fim de evitar complicações de saúde e melhorar a qualidade de vida da criança e de sua família^(10)^.

Para o diagnóstico de DA, vários instrumentos estão disponíveis com uma grande heterogeneidade: alguns baseados em questionários, outros em observação direta da deglutição e que avaliam desde a sensibilidade oral até as habilidades motoras orais da criança^(11)^. A Escala Brasileira de Alimentação Infantil (EBAI), instrumento de triagem que permite avaliação abrangente diante das dimensões biopsicossociais que envolvem a DA, é um instrumento validado transculturalmente, composto de 14 perguntas, autoaplicável, que avalia a gravidade da DA e sete domínios, a saber: motor-oral, sensório-oral, apetite e outros quatros relacionados à condições psicossociais (preocupação materna com a alimentação, comportamento da criança nas refeições, estratégias maternas na refeição e reações familiares à alimentação da criança). Diante dessas características, a EBAI se apresenta como um instrumento de rápida aplicação e útil para os profissionais que desejam triar recém-nascidos para DA^(12)^.

O objetivo deste estudo é descrever a prevalência de dificuldades alimentares em crianças com idade entre seis meses e seis anos e onze meses, nascidas prematuras, e analisar as relações com as condições perinatais e neonatais pesquisadas.

## MÉTODO

Este é um estudo ambispectivo transversal, aprovado pelo Comitê de Ética em Pesquisa da instituição sob número CAAE: 65887622.1.0000.0096.

A pesquisa foi realizada com 129 pais/cuidadores de crianças nascidas prematuras, alimentadas com dieta sólida ou pastosa, após assinarem o Termo de Consentimento Livre e Esclarecido no momento da consulta nos ambulatórios de prematuros de um hospital terciário, de fevereiro de 2023 a janeiro de 2024. No ambulatório, 152 crianças elegíveis foram acompanhadas, e o cálculo amostral indicou que, com grau de confiança de 95% e margem de erro de 5%, a amostra mínima seria 110 indivíduos. Foram selecionadas por conveniência 129 crianças (Figura 1). Nenhuma das crianças participantes havia recebido tratamento fonoaudiológico.

**Figura 1:** População de estudo

Como prematuras, considerou-se crianças nascidas com menos que 37 semanas de idade gestacional, determinada por ecografia precoce^(13)^ ou método New Ballard^(14)^, classificadas em quatro grupos: prematuros tardios (de 34 a 36 semanas(s) e 6 dias (d)), prematuros moderados (32s a 33s e 6d), muito prematuros (28s a 31s e 6d) e prematuros extremos (< 28s)^(15,16)^. A população que participou efetivamente consistiu em crianças de seis meses de idade corrigida até cinco anos e sete meses.

Após a concordância, os cuidadores foram convidados ao autopreenchimento da Escala Brasileira de Alimentação Infantil (EBAI)^(12)^. Os dados perinatais e neonatais foram coletados nos prontuários. As variáveis obtidas nos prontuários foram: sexo da criança; idade gestacional; idade no dia da aplicação; peso ao nascer; idade cronológica na alta; tempo de intubação orotraqueal (IOT); dias de suporte ventilatório; tempo de fototerapia; grau de prematuridade; diabetes gestacional (DMG); doença hipertensiva específica da gravidez (DHEG); tipo de parto; escore de APGAR no primeiro e quinto minuto; presença de icterícia; e peso ao nascimento para a idade gestacional, conforme gráfico de Fenton^(17)^ — Pequeno para Idade Gestacional (PIG), Adequado para a Idade Gestacional (AIG) e Grande para a Idade Gestacional (GIG)^(18)^.

Como critérios de inclusão, considerou-se: famílias com crianças com idade corrigida entre seis meses e seis anos e onze meses, prematuras, avaliadas por ecografia precoce (antes de 13 semanas e seis dias de gestação ou na ausência desta, pelo método New Ballard e concordância na participação da pesquisa pelos cuidadores.

Como critérios de exclusão, considerou-se: malformações congênitas complexas, uso de vias alternativas de alimentação, não ter iniciado a introdução alimentar, crianças não neurotípicas e falta de dados críticos nos prontuários (como peso ao nascer e idade gestacional).

### Escala Brasileira de Alimentação Infantil (EBAI)

A EBAI é um instrumento de triagem composto por 14 perguntas, e pode ser preenchido fácil e rapidamente pelos pais (autoaplicável). Foi validada transculturalmente para o Brasil em 2021^(12)^, a partir da *Montreal Children’s Hospital Feeding Scale* (MCH-FS)^(19)^. O preenchimento da EBAI resulta em quatro categorias: sem DA, DA leve, DA moderada e DA grave. Os 14 itens abrangem sete domínios com sobreposições: domínio motor-oral (itens 8 e 11), sensório-oral (itens 7 e 8), apetite (itens 3 e 4), preocupação materna com a alimentação (itens 1, 2 e 12), comportamento na hora das refeições (itens 6 e 8), estratégias maternas utilizadas (itens 5,9 e 10) e reações familiares à alimentação da criança (itens 13 e 14), sendo que estes quatro últimos domínios compreendem a dimensão psicossocial. Cada item é pontuado numa escala Likert de sete pontos. Das 14 perguntas, sete seguem direção de negativo para positivo (sendo 1 mais grave e 7 sem problema) e sete seguem direção do positivo para o negativo. A mãe ou cuidador que alimenta a criança marca cada item de acordo com a frequência em que ocorre, ou o grau de dificuldade que percebe determinado comportamento ou o nível de preocupação que apresenta em relação à pergunta. Após a somatória dos pontos, encontra-se o Escore Bruto que é convertido numa tabela em Escore T, que, se igual ou maior que 61, indica triagem positiva para DA. O questionário pode ser preenchido em aproximadamente cinco minutos. Para a pontuação, os itens que seguem a sequência de negativo para positivo são invertidos pelo pesquisador que obtém o escore bruto, e o converte numa tabela em Escore T. Nessa tabela, a dificuldade é classificada nas quatro categorias^(12)^.

### Análise estatística

Os dados foram organizados em planilha Excel^®^ e analisados com o programa computacional IBM SPSS Statistics v.29.0. Resultados de variáveis quantitativas foram descritos por média, desvio padrão, mediana, mínimo e máximo. Variáveis categóricas (sexo, grau de prematuridade, DMG, DHEG, tipo de parto, escore de APGAR categorizado, icterícia e peso para idade gestacional) foram descritas por frequência absoluta e percentual. As comparações de dois grupos definidos por variáveis relativas à DA (não ou sim) e por tipo de parto (vaginal ou cesárea), em relação a variáveis quantitativas (idade gestacional, idade na aplicação do questionário, peso ao nascer, idade na alta, tempo de IOT, dias de suporte ventilatório e tempo de fototerapia), foram feitas usando-se o teste t de Student para amostras independentes ou o teste não-paramétrico de Mann-Whitney. Os grupos definidos por prematuridade (tardio, moderado, muito prematuro ou extremo) e por AIG/PIG/GIG foram comparados em relação a variáveis quantitativas usando-se o teste não-paramétrico de Kruskal-Wallis e o teste post-hoc de Dunn. Variáveis categóricas foram analisadas usando-se o teste exato de Fisher ou o teste de Qui-quadrado. Para avaliação da correlação entre duas variáveis quantitativas, estimou-se coeficientes de correlação de Spearman. A condição de normalidade de variáveis quantitativas foi avaliada pelo teste de Kolmogorov-Smirnov. Valores de p < 0,05 indicaram significância estatística. Para comparações múltiplas de grupos, os valores de p foram corrigidos por Bonferroni.

## RESULTADOS

Na população de 129 crianças, 52,7% eram do sexo feminino e 47,3% do sexo masculino. O peso ao nascer variou de 540g até 3585g. Em maioria, os indivíduos foram classificados como Prematuros Tardios, seguidos em quantidade pelos Muito Prematuros, Prematuros Moderados e, em menor quantidade, Prematuros Extremos (Tabela 1). Na data da aplicação, a idade média das crianças era de 1,8 anos, com mediana de 1,7 (mínimo de 0,6 anos e máxima de 5,6 anos)

**Tabela 1:** Variáveis perinatais e neonatais e relação com dificuldade alimentar

**Variável**	**Classificação**	**n**	**Grupo**		**p** ^*^	**OR (IC95%)**
			**Normal**	**Dificuldade alimentar**		
PESO AO NASCER PARA A IDADE GESTACIONAL	GIG (ref)	7	7 (100%)	0 (0%)		
	AIG	95	87 (91,6%)	8 (8,4%)	00,300	-
	PIG	27	20 (74,1%)	7 (25,9%)	00,022	-
DMG	Não	102	87 (83%)	15 (14,7%)		
	Sim	27	27 (100%)	0 (0%)	00,040	-
Fototerapia	Não	40	30 (75%)	10 (25%)		
	Sim	88	83 (94,3%)	5 (5,7%)	00,004	0,18 (0,06 - 0,57)
ICTERÍCIA	Não	29	23 (79,3%)	6 (20,7%)		
	Sim	99	90 (90,9%)	9 (9,1%)	00,096	0,38 (0,12 - 1,19)
Idade na aplicação da EBAI (anos)	[média ± dp	129	1,8 ± 0,9	1,9 ± 0,8	00,587	1,16 (0,68 – 2,00)
	(min-max)]		(0,6 - 5,6)	(0,7 - 4,3)		
Dias internado	[mediana	129	30,5	23	00,598	1,00 (0,98 - 1,01)
	(min-max)]		(3 - 173)	(3 - 113)		
IG (semanas)	[média ± dp	129	32 ± 3,2	33 ± 2,7	00,282	1,11 (0,92 - 1,34)
	(min-max)]		(24 - 36,9)	(25,6 - 36,7)		
Peso ao nascimento (g)	[média ± dp	129	1705 ± 667	1599 ± 562	00,558	1 (0,999 - 1,001)
	(min-max)]		(540 - 3585)	(859 - 2830)		
Tempo de IOT (dias)	[mediana	129	0 (0 - 48)	0 (0 - 17)	00,391	0,95 (0,85 - 1,06)
	(min-max)]					
Total de dias com algum suporte respiratório	[mediana	129	5 (0 - 107)	3 (0 - 85)	00,810	1 (0,97 - 1,02)
	(min-max)]					
Tempo de fototerapia (dias)	[mediana (min-max)]	128	2 (0 - 10)	0 (0 - 5)	00,040	0,63 (0,40 - 0,98)
Prematuridade	Tardio (ref)	44	39 (88,6%)	5 (11,4%)		
	Moderado	32	25 (78,1%)	7 (21,9%)	00,222	2,18 (0,62 - 7,64)
	Muito prematuro	36	34 (94,4%)	2 (5,6%)	00,370	0,46 (0,08 - 2,52)
	Extremo	17	16 (94,1%)	1 (5,9%)	00,527	0,49 (0,05 - 4,51)
DHEG	Não	94	86 (91,5%)	8 (8,5%)		
	Sim	34	27 (79,4%)	7 (20,6%)	00,068	2,79 (0,93 - 8,40)
TIPO DE PARTO	Vaginal	44	42 (95,5%)	2 (4,5%)		
	Cesárea	83	70 (84,3%)	13 (15,7%)	00,083	3,90 (0,84 - 18,1)
Escore de APGAR 1 min	(7 a 10) (ref)	80	68 (85,0%)	12 (15,0%)		
	(4 a 6)	37	35 (94,6%)	2 (5,4%)	00,154	0,32 (0,07 - 1,53)
	(0 a 3)	12	11 (91,7%)	1 (8,3%)	00,543	0,52 (0,06 - 4,37)
Escore de APGAR 5min	(7 a 10)	117	103 (88,0%)	14 (12,0%)		
	(4 a 6)	11	10 (90,9%)	1 (9,1%)		
	(0 a 3)	1	1	0	-	-
Sexo	Feminino	68	62 (91,2%)	6 (8,8%)		
	Masculino	61	52 (85,2%)	9 (14,8%)	00,283	1,80 (0,61 - 5,46)

p < 0,05

* Modelo de Regressão Logística e teste de Wald ou teste exato de Fisher

**Legenda:** DMG= diabetes mellitus gestacional; DHEG=doença hipertensiva específica da gestação; PIG= pequeno para idade gestacional; AIG= adequado para idade gestacional; GIG= grande para a idade gestacional; IOT=intubação orotraqueal; ref=variável de referência; min= minuto; g=grama; OR: odds ratio; IC95%: intervalo de confiança de 95%; min= valor mínimo; máx= valor máximo; dp= desvio padrão; IG = Idade Gestacional.

### Prevalência de Dificuldades Alimentares em Crianças prematuras

Das 129 crianças que participaram, 15 apresentaram DA, o que mostra prevalência de 11,62%. Entre essas crianças, seis (40%) apresentaram DA leve, sete (47,7%) DA moderada e duas (13,3%) DA grave.

### Relação entre as variáveis perinatais e neonatais e DA

Apenas três das variáveis independentes estudadas mostraram relação significativa com DA (p < 0,05): classificação do peso ao nascimento para a idade gestacional, Diabetes Mellitus Gestacional e uso de fototerapia. As demais não apresentaram significância estatística.

#### Classificação do peso ao nascimento para a idade gestacional

Na população estudada, 73,64% das crianças nasceram AIG, 20,93% PIG e 5,42% GIG. Entre os GIG, nenhuma criança apresentou DA e a maior incidência ocorreu nos PIG.

A prevalência de DA em PIG é significativamente maior que nas outras categorias de peso ao nascer (Tabela 1).

A população de PIG foi menor que a de AIG, porém quase metade das crianças com DA eram PIG. Essa proporção foi significativa estatisticamente (Tabela 2).

**Tabela 2:** Proporção de indivíduos com e sem DA e Peso para idade gestacional

	n	PIG	AIG	GIG
SEM DA	114	20(17,5%)	87(76,4%)	7(6,1%)
DA	15	7 (46,7%)	8(53,3%)	0(0,0%)
TOTAL	129	27	95	7
		p=0,023	p=0,112	p=0,703

Legenda: n=número de sujeitos; PIG=pequeno para idade gestacional; AIG=Adequado para idade gestacional; GIG=grande para a idade gestacional; DA=dificuldade alimentar.

#### Diabetes Mellitus Gestacional (DMG) e fototerapia

Entre as crianças com mãe com DMG, nenhuma apresentou DA. Todas as crianças com DA nasceram de mães sem DMG, o que mostrou significância estatística.

A presença de DA foi menor naquelas que fizeram fototerapia e também naquelas com maior tempo de fototerapia. Essa diferença mostrou-se significativa estatisticamente.

O tempo de fototerapia foi analisado em relação aos grupos AIG, PIG e GIG, sendo que esse tempo foi bastante menor na população PIG quando comparado com os outros dois grupos (Tabela 3).

**Tabela 3:** Tempo de fototerapia e peso ao nascer para a idade gestacional

**Variável**	**AIG/PIG/GIG**	**n**	**Média**	**Desvio padrão**	**Mediana**	**Mínimo**	**Máximo**	**p** ^*^
Tempo de fototerapia (dias)	AIG	94	2,1	2,1	2	0	10	
	PIG	27	1,0	1,4	0	0	5	
	GIG	7	2,9	2,8	2	0	8	0,014

p < 0,05

* Teste não-paramétrico de Kruskal-Wallis

Também foi analisado o coeficiente de correlação de Spearman, que mostrou correlação inversa, porém fraca, entre o tempo de fototerapia e todos os domínios avaliados pela EBAI, com correlação inversa e significativa no domínio Sensório-Oral. Já o tempo de suporte ventilatório mostrou relação direta e significativa com o domínio Motor Oral (Tabela 4). As outras variáveis estudadas não mostraram significância estatística com nenhum dos domínios da EBAI.

**Tabela 4:** Correlação entre tempo de fototerapia e de suporte ventilatório com os domínios avaliados na EBAI

	n	r	p
Tempo de fototerapia (dias) x T-score	128	-0,10	0,240
Tempo de fototerapia (dias) x Motor-oral	128	-0,05	0,573
Tempo de fototerapia (dias) x Sensório-oral	128	-0,25	0,005
Tempo de fototerapia (dias) x Apetite	128	-0,12	0,172
Tempo de fototerapia (dias) x Preocupação materna	128	-0,17	0,053
Tempo de fototerapia (dias) x Comportamento nas refeições	128	-0,15	0,093
Tempo de fototerapia (dias) x Estratégias familiares	128	-0,05	0,543
Tempo de fototerapia (dias) x Reações dos familiares	128	-0,10	0,240
Tempo com algum suporte ventilatório (dias) x T-score	129	0,14	0,125
Tempo com algum suporte ventilatório (dias) x Motor-oral	129	0,18	0,045
Tempo com algum suporte ventilatório (dias) x Sensório-oral	129	0,03	0,698
Tempo com algum suporte ventilatório (dias) x Apetite	129	-0,01	0,908
Tempo com algum suporte ventilatório (dias) x Preocupação materna	129	0,05	0,585
Tempo com algum suporte ventilatório (dias) x Comportamento nas refeições	129	0,08	0,347
Tempo com algum suporte ventilatório (dias) x Estratégias familiares	129	-0,02	0,817
Tempo com algum suporte ventilatório (dias) x Reações dos familiares	129	0,14	0,125

**Legenda:** n=número de indivíduos r= Coeficiente de Spearman; n=número de indivíduos; T-score= escore da Escala Brasileira de Alimentação Infantil

Não foram encontradas associações significativas entre as demais variáveis estudadas e dificuldades alimentares.

## DISCUSSÃO

Esta pesquisa mostrou que a DA, avaliada pela EBAI, é relativamente baixa quando comparada à maioria dos trabalhos pesquisados. Em uma metanálise que avaliou 22 estudos, com 4381 prematuros de todas as IG e que utilizaram vários métodos de avaliação como escalas formais, informais ou clínicas aplicadas até 48 meses de idade, Pados *et al*. estimam que 42% das crianças prematuras possam apresentar algum tipo de DA, sem prevalência significativa entre as categorias de idade gestacional ou idade da avaliação^(5)^. Em revisão crítica de 22 estudos, com 3149 crianças, a partir da aplicação de questionários estruturados ou observação direta, Pagliaro *et al*. estimam que entre 25% e 80% dos prematuros podem apresentar DA^(6)^. A prevalência encontrada neste trabalho foi de 11,62% (n=15), valor muito próximo ao encontrado (11%) por Nieuwenhuis *et* *al*. ao aplicar o questionário *Screeningslijst Eetgedrag Peuters* (SEP), instrumento de triagem validado na Holanda a partir do mesmo questionário original usado nesta pesquisa, o *MCH-FS*. No mesmo trabalho, esse autor comparou 30 prematuros com 248 não prematuros aos três anos de idade e não encontrou diferenças significativas entre os PIG prematuros e não prematuros^(20)^.

Neste trabalho, nascer PIG mostrou ser estatisticamente significativo na determinação de DA, visto que 25,92% dos indivíduos apresentaram DA contra apenas 8,42% dos AIG e nenhum dos GIG.

O nascimento PIG, principalmente se for prematuro, predispõe a problemas de desenvolvimento neurológico, com potencial risco de disfunção neurológica não severa, problemas cognitivos e de atenção e baixas competências sociais^(21)^, tendo em vista que, no momento da refeição, ocorre grande interação da criança com a mãe ou cuidadores e exige da criança capacidades motoras, sensoriais e cognitivas^(10)^. É possível que as consequências sobre o neurodesenvolvimento, mesmo sutis, possam favorecer o aparecimento de DA.

Nenhuma das crianças cujas mães apresentaram DMG pontuaram para DA. Apesar de a DMG ser fator predisponente para crianças nascerem GIG^(22)^, podendo chegar de 20 a 30%^(23)^, apenas dois (7,40%) dos filhos de mães com DMG nasceram GIG, a maioria (74%) foram AIG. É possível que a alta prevalência de AIG tenha ocorrido devido ao controle adequado dessa condição no pré-natal, visto que essas mães fizeram seus acompanhamentos em hospital terciário e universitário. Na literatura, não foi encontrada nenhuma referência à possível relação negativa entre nascer prematuro de mãe DMG e DA. Ao contrário, estudos evidenciam efeitos negativos da DMG materna a curto e longo prazo, tanto na saúde física dos filhos dessas mães com prejuízos do neurodesenvolvimento e da cognição^(24,25)^. Esse grupo teve prevalência baixa de PIG (18,6%), e PIG foi o grupo com maior prevalência de DA — esse dado pode ter sido a causa do resultado encontrado.

O grupo submetido à fototerapia também teve menos DA. A explicação para esse achado não é clara e não foi encontrada na literatura relação negativa entre a fototerapia e DA. Entretanto, crianças extremamente prematuras expostas à fototerapia apresentam menor comprometimento neurológico que aquelas não expostas^(26)^. Outra possível explicação para esse achado é que a DA foi mais prevalente em PIG e estes tiveram tempo de fototerapia significativamente menor em relação aos AIG e GIG. Dessa maneira, pode-se ter confundido as correlações, ou seja, se AIG e GIG diminui o risco de apresentar DA, como estes grupos fizeram mais fototerapia, pode parecer que o tempo de fototerapia tem relação inversa com aparecimento de DA.

Como não há respaldo na literatura pesquisada, a relação negativa entre DMG e fototerapia com DA precisa ser mais bem estudada, pois pode ter sido um achado eventual.

Avaliou-se também o coeficiente de correlação de Spearman, que mostrou correspondência fraca entre a maioria das variáveis em relação aos domínios analisados pela EBAI. Vale citar que o tempo de fototerapia teve relação inversa, porém fraca, com o prejuízo nos diversos domínios, mas teve relação estatisticamente significativa com o domínio Sensório Motor, resultado que não encontra referências na literatura e precisa ser melhor avaliado. Já o tempo de suporte ventilatório se correlacionou fraca e diretamente, mas de forma significativa, com a piora do domínio Motor-Oral, e encontra respaldo na literatura, porém sem explicação para o achado^(7,27)^ .

Apesar do grau de prematuridade ser citado como causa de DA^(7)^, neste trabalho não foram observadas diferenças significativas entre os diferentes graus de prematuridade. Isso pode ter ocorrido porque o aparecimento de DA é determinado por vários fatores e não apenas a prematuridade isoladamente^(28)^. Outra possibilidade é que, ao longo do tempo, a disfunção motora oral pode diminuir em sua frequência em decorrência do crescimento e do desenvolvimento psicomotor do nascido prematuro^(27)^.

Este estudo apresenta algumas limitações. A maioria das crianças foram consideradas prematuras de risco ao nascer e necessitaram de atendimento intensivo, e, como consequência, receberam atendimento em ambulatório terciário. Estudos comparativos entre prematuros menos graves e com não-prematuros poderiam apresentar resultados diferentes em relação a prevalência e fatores de risco e proteção.

As diferenças entre a literatura e este trabalho podem ser explicadas pelo uso de metodologias diferentes: observação direta da alimentação^(29)^, entrevista semiestruturada com os pais^(30)^ e questionário diferente da EBAI^(31)^. Geralmente, os instrumentos utilizados avaliam um domínio específico e a EBAI, por ser um instrumento de triagem de abrangência integral, inclui aspectos sensoriais, motores, de apetite e psicossociais^(12)^.

Por fim, a busca de informações retrospectivamente nos prontuários esbarra, algumas vezes, em falta de padronização das informações ou mesmo falta delas. Um estudo prospectivo, de longo prazo, com número maior de pacientes, poderia ser delineado para uma compreensão mais adequada das DA em prematuros.

## CONCLUSÃO

O estudo, utilizando um instrumento validado (EBAI), determinou a prevalência de Dificuldades Alimentares em 11,62% da população de prematuros estudados. Prematuros Pequenos para a Idade Gestacional apresentaram mais DA que os prematuros AIG e GIG. Prematuros submetidos à fototerapia, aqueles com maior tempo de fototerapia e os filhos de mães com DMG apresentaram menos DA. As outras variáveis perinatais e neonatais não mostraram força estatística em relação à DA.
